# Thermally Activated Photophysical Processes of Organolanthanide Complexes in Solution

**DOI:** 10.1021/acs.jpclett.2c01350

**Published:** 2022-05-26

**Authors:** Waygen Thor, Hei-Yui Kai, Yonghong Zhang, Ka-Leung Wong, Peter A. Tanner

**Affiliations:** † Department of Chemistry, Hong Kong Baptist University, Waterloo Road, Kowloon Tong, Hong Kong S.A.R., P. R. China; ‡ State Key Laboratory of Chemistry and Utilization of Carbon Based Energy Resources, Key Laboratory of Oil and Gas Fine Chemicals, Ministry of Education & Xinjiang Uygur Autonomous Region, Urumqi Key Laboratory of Green Catalysis and Synthesis Technology, College of Chemistry, Xinjiang University, Urumqi 830046, Xinjiang, P. R. China

## Abstract

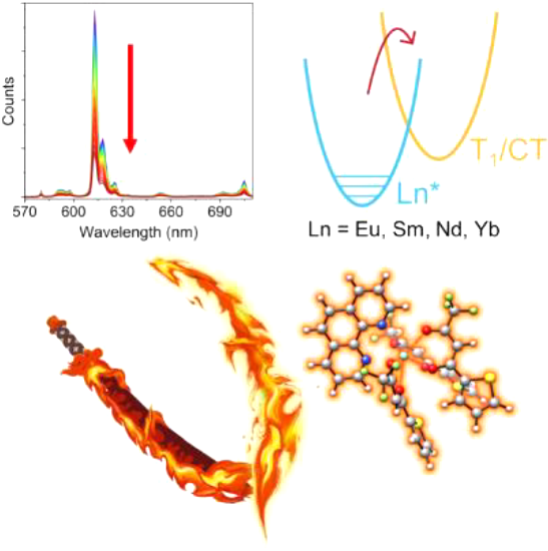

The effect of temperature upon the lanthanide luminescence lifetime and intensity has been investigated in toluene solution for the complexes **LnPhen(TTA)**_**3**_ (Ln = Eu, Sm, Nd, Yb; Phen = 1,10-phenanthroline; TTA = thenoyltrifluoroacetonate). Thermally excited back-transfer to a charge transfer state was found to occur for Ln = Eu and can be explained by lifetime and intensity back-transfer models. The emission intensity and lifetime were also quenched with increasing temperature for Ln = Sm, and the activation energy for nonradiative decay is similar to that for the thermal population of Sm^3+^ excited states. Unusual behavior for lifetime and intensity was found for both Ln = Nd, Yb. The usually assumed equivalence of τ/τ_0_ = *I*/*I*_0_ (where τ is lifetime and *I* is intensity) does not hold for these cases. We infer that for these lanthanide systems the intensity decreases with temperature in the stage prior to population of the luminescent state. The lifetime changes are discussed.

For many decades, the unique sharp emission bands and long lifetimes of trivalent lanthanide ions (Ln^3+^) have received attention in diverse fields of application,^1^ including light emitting diodes (LEDs)^2,3^ and imaging probes for the biomedical field.^4,5^ More recently, the use of Ln^3+^ ions in thermal sensing, employing homonuclear^6,7^ or heteronuclear^8−10^ complexes, has been a burgeoning field of study. To further this application, the mechanism of thermal quenching needs to be thoroughly investigated and understood. This present work utilizes different Ln^3+^ ions in the same organometallic complex in order to understand and compare the temperature quenching mechanisms.

In organolanthanide complexes, the sensitization of Ln^3+^ is often taken to follow the S_0_ → S_1_ → T_1_ → Ln^3+^ pathway,^11,12^ although other mechanisms have been put forward. Emission from a lanthanide ion is observed when the excited state relaxes with the radiative rate *k*_r_ and nonradiative rate *k*_nr_, with *k*_r_ > *k*_nr_. The increase of nonradiative rate with temperature may be due to (i) increased vibrational relaxation to a lower state,^13^ (ii) thermally induced energy transfer,^14^ or (iii) electron transfer processes.^15^ In addition, other processes such as those affecting excitation may change the intensity of emission. It is important to distinguish temperature-dependent and -independent nonradiative losses—whether the quenching occurs before or after the excitation reaches the luminescent state—and this may be possible by comparing emission intensity with lifetime measurements to see if they follow a similar trend.

The emission quenching can be modeled by a simple Arrhenius process with activation energy *E* to a higher state such as a triplet in the case of Tb^3+^.^16^ Alternatively, a scenario for thermal quenching of luminescence lifetime τ or intensity *I* due to a single-barrier back-transfer to a triplet state, conduction band,^17^ or a charge-transfer (CT) state^18,19^ (Figure 1a) has been modeled by eqs 1 and 2
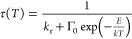
1and assuming that τ(*T*)/τ_r_ = *I*(*T*)/*I*_0_, where τ_r_ = 1/*k*_r_^20^
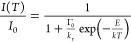
2where Γ_0_ is the attempt nonradiative rate, *k* is the Boltzmann constant, and *T* is the temperature.

**Figure 1:**
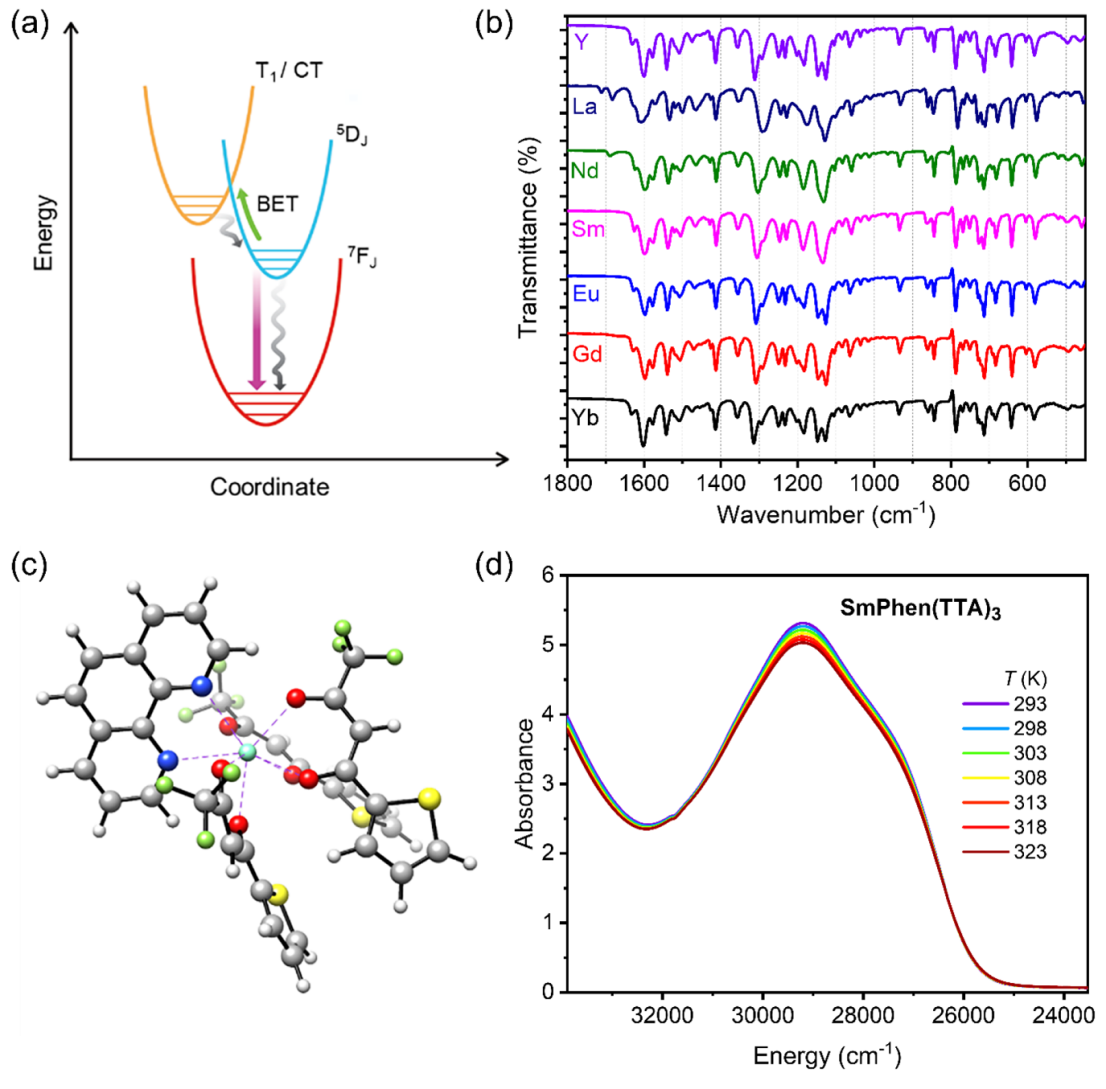
(a) Illustration of the thermally activated single-barrier back-energy transfer (BET) using Eu^3+^ as an example. (b) FTIR spectra of **LnPhen(TTA)**_**3**_, Ln = Y, La, Nd, Sm, Eu, Gd, and Yb. (c) Optimized structure of **SmPhen(TTA)**_**3**_ using the PBE0/def2-TZVP level of theory. C: gray; H: white; N: blue; O: red; F: green; S: yellow; Sm: Cyan. (d) Absorption spectra of 10 μM SmPhen(TTA)_3_ in toluene at different temperatures corrected with the thermal expansion of toluene (0.00108 mL/K).

Various strategies have been applied to optimize the antenna design for the triplet state in light-harvesting for lanthanide ions. These include the chemical variation of the triplet state energy of the lanthanide complex and the tuning of the location of the CT energy level. Since eqs 1 and 2 describe single-barrier back-energy transfer, the location of the triplet state or the CT state energy level can in principle be estimated using thermal quenching experiments.

In this work, by using the same complex **LnPhen(TTA)**_**3**_ (Phen = 1,10-phenanthroline; TTA = thenoyltrifluoroacetonate), we enquire how the mechanisms of thermal quenching properties vary for different Ln^3+^ ions. The relevant energy level diagrams for the ions are included in Figure S1. The luminescence lifetime and intensity have been recorded at different temperatures for each complex dissolved at low concentration in toluene solution. We have chosen this complex because it is well-researched^21^ and gives efficient luminescence for Ln^3+^.^22−24^ The materials, syntheses, and instrumental details are presented in the Supporting Information.

Our complexes have been characterized spectroscopically (Figure 1b) and by DFT^25^ (Figure 1c). The FTIR spectra of solid **LnPhen(TTA)**_**3**_ (Figure 1b) are similar and show that the coordination of the antennae to the Ln^3+^ does not change across the series. The calculated^25^ vibrational frequencies of **SmPhen(TTA)**_**3**_ are in good agreement with the experimental data (Figure S2). The electronic absorption spectra of **LnPhen(TTA)**_**3**_ are similar across the series, with a peak maximum at ∼340 nm and a shoulder at ∼360 nm (Figure S3a). A minor blue shift of the absorption maximum can be observed with a decreasing ionic radius of Ln^3+^ (Figure S3b). The absorption spectra do not exhibit remarkable wavelength or absorbance shifts with temperature (for example, as in Figures 1d and S4). Such shifts may account for luminescence intensity quenching when using a fixed excitation wavelength.

Generally, the luminescence intensity of **LnPhen(TTA)**_**3**_ (Ln = Eu, Sm, Yb, and Nd) decreases with increasing temperature. The ligand singlet level lies below the lanthanide energy levels for Ln = Y, La, and Gd, and hence, ligand–metal ion energy transfer does not occur in those cases. We now discuss the results from individual lanthanide ions in turn, starting with Eu^3+^, for which the ^5^D_0_ level is situated at more than 11 900 cm^–1^ above the next-lowest level, ^7^F_6_, so that multiphonon relaxation is slow. The dominant mechanism for temperature quenching should then involve back-energy transfer to an excited state. The variation of the emission intensity of **EuPhen(TTA)**_**3**_ with temperature is shown in Figure 2a, using 342 nm excitation into the ligand absorption band. The luminescence decay curves using 355 nm excitation are shown in Figure 2b. Over the temperature range from 283 to 333 K, the intensity decreases by a factor of 3.8, whereas the emission lifetime decreases by the factor of 2.1. Figure S5 shows that the lifetime and intensity data follow the same trend only after about 305 K so that then both apply to processes occurring solely in the ^5^D_0_ state. Our measurement of the emission lifetime at 77 K yields 0.708 ms, and this leads to the estimated value of *k*_r_ = 1412 s^–1^. We have treated the data in Figure 2 assuming temperature-assisted back-energy transfer from ^5^D_0_ to a single upper state. Using the above value of *k*_r_, the value of activation energy from a three-parameter fit to the intensity data using eq 2 is displayed in the inset of Figure 2a, with fits to the entire data set (red) or to the data points above 303 K (blue), which show a similar trend to lifetime data in Figure S5a. The correction for the volume expansion of toluene has a negligible effect upon the fits and parameters and is neglected here and subsequently. The fit to the lifetime data using eq 1 with three parameters gives the values *E* = 5282 ± 101 cm^–1^, Γ_0_ = (1.4 ± 0.6) × 10^13^ s^–1^, and *k*_r_ = 1460 ± 2 s^–1^, with the latter value not far from the measured 77 K value. Considering the additional back-energy transfer from ^5^D_0_ to the ^5^D_1_ state situated at 1746 cm^–1^ higher energy, two-level back-transfer fits are discussed in the Supporting Information, section E, and found to be inapplicable. In conclusion, the back-energy transfer is directed dominantly to one excited level situated about 5000 cm^–1^ above ^5^D_0_.

**Figure 2:**
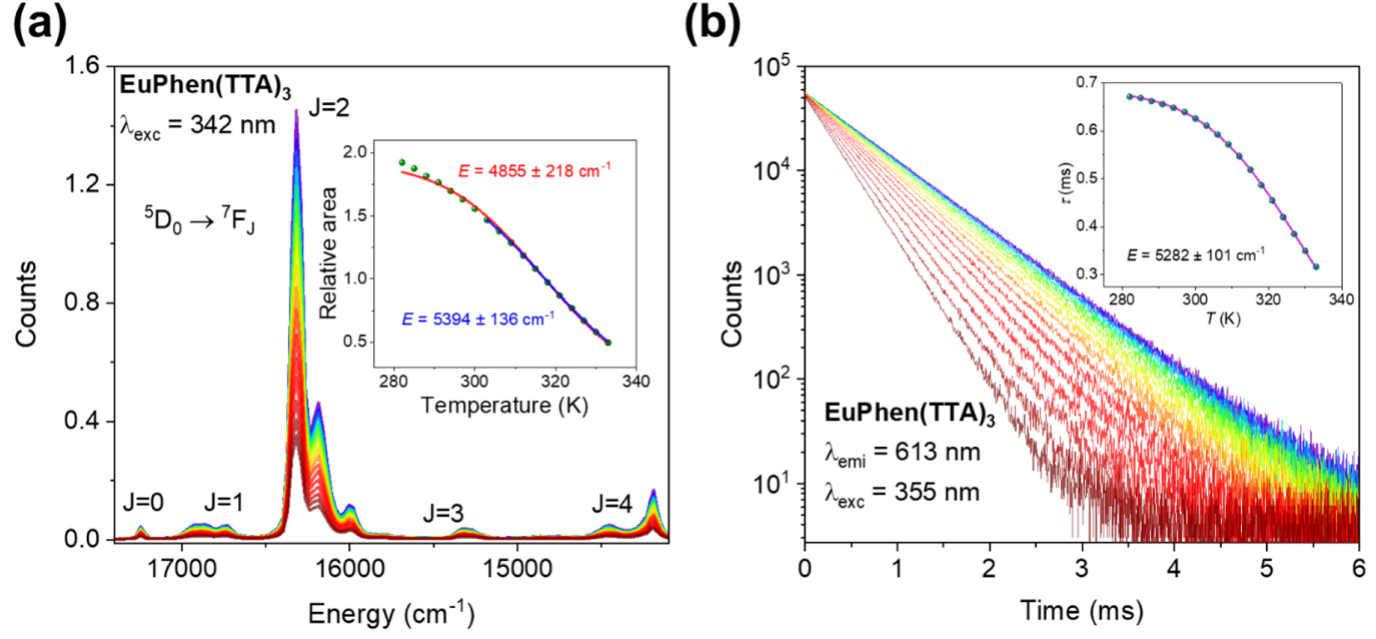
(a) Evolution of the emission spectrum of 10 μM **EuPhen(TTA)**_**3**_ in toluene with respect to temperature. The inset shows the integrated area of the ^5^D_0_ → ^7^F_2_ Eu^3+^ emission with temperature after a Jacobian correction of each spectrum to an energy scale. Alternative fittings using eq 2 with the value *k*_r_ = 1412 s^–1^ are displayed in red and blue in the inset. (b) Evolution of the lifetime of 10 μM **EuPhen(TTA)**_**3**_ in toluene with respect to temperature. The inset shows the lifetime variation of Eu^3+^ with temperature, and the fit using eq 1 is displayed in purple. In both figures, purple represents the lowest temperature (282 K), and red represents the highest temperature (333 K) with steps of 3 K.

The nature of this excited level is now considered. Figure S7 shows that for Ln = Gd, Y, and La, the ligand triplet state is located at ∼488 nm (20 480 ± 50 cm^–1^). The energy gap from ^5^D_0_ at 17242 cm^–1^ is only 3238 cm^–1^, which is considerably smaller than the calculated activation energy. Back-energy transfer to ^5^D_2_ (at 4180 cm^–1^) is also discounted. Berry et al.^26^ have attributed the back-energy transfer in solid-state europium tris(2,2,6,6-tetramethyl-3,5-heptanedionato) (hereafter abbreviated to Eu(thd)_3_) to a charge transfer state. We plot their data according to the simple Arrhenius model in Figure S8a. The ^5^D_0_ nonradiative rate is very small and constant up to 236 K, and then, it increases. We fitted the rising linear portion to give the activation energy of 4076 ± 41 cm^–1^. There is no evidence for dominant back-transfer to a second state above the temperature of 290 K. The analogous plot of our lifetime data is presented in Figure S8b, and a linear fitting with an activation energy of 4712 ± 50 cm^–1^ is obtained above 297 K. The portion to lower temperature in this figure exhibits a lower activation energy (roughly 2580 ± 166 cm^–1^ from five data points), just like the portion between 236–290 K in Figure S8a. Presumably, the lower activation energy in this temperature range corresponds to back-transfer to ^5^D_1_ and to the triplet state.

Generally, although other parameters may be involved, the charge transfer energy can be related to the electric dipole/magnetic dipole ratio of the emission spectrum, since the ^5^D_0_ → ^7^F_2_ Eu^3+^ emission intensity can be enhanced, whereas ^5^D_0_ → ^7^F_1_ is not, to first order. The degree of enhancement is inversely proportional to the energy separation of the CT band from that of ^5^D_0_. Taking the ratio (^5^D_0_ → ^7^F_2_)/(^5^D_0_ → ^7^F_1_) = *R* as an illustration, the value of *R* for Eu(thd)_3_ is 19.9, whereas it is 13.0 for **EuPhen(TTA)**_**3**_. The charge transfer band is therefore expected at higher energy in the present case than for Eu(thd)_3_.

The charge transfer vertical transition state for **EuPhen(TTA)**_**3**_ is estimated at 385 nm (25 974 cm^–1^) from the subtraction of the **EuPhen(TTA)**_**3**_ and **GdPhen(TTA)**_**3**_ electronic absorption spectra in Figure S9. As noted by Blasse et al.,^27^ the location of the vertical charge transfer transition energy may differ appreciably from that of the intersection of the 4f^6 7^F_*J*_ level manifold with the charge transfer state given by *E*.

We move on to the quenching of Sm^3+^ emission in both the visible (below 565 nm) and near-infrared (below ∼880 nm) regions, which occurs from the same luminescent state, ^4^G_5/2_ at ∼17 700 cm^–1^. The emission spectra of **SmPhen(TTA)**_**3**_ in toluene are displayed in Figure 3a,b as a function of temperature. The charge transfer state of **SmPhen(TTA)**_**3**_ is calculated to lie at 9103 ± 1231 cm^–1^ above that of **EuPhen(TTA)**_**3**_(28) so that back-energy transfer to this state does not occur at the temperatures investigated. The next-lowest level, at ∼9200 cm^–1^ below ^4^G_5/2_, is ^6^F_9/2_,^29^ and using the equation for the temperature dependence of multiphonon relaxation rate for weak coupling
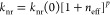
3a
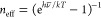
3bwhere ν̅ is an effective phonon frequency and *p* is the order of the process, it is not possible to obtain a data fit using sensible values for ν̅ and *p*. The ^4^F_3/2_ and ^4^G_7/2_ levels of Sm^3+^ are situated at ∼1000 and ∼2000 cm^–1^ above ^4^G_5/2_, respectively,^30,31^ so that, just as for the following case of Nd^3+^ subsequently discussed, the multiphonon decay rates from these states to ^4^G_5/2_ are very high. The Arrhenius plot of the variation of nonradiative rate with temperature (Figure S10) shows that the activation energy changes with temperature, with the slope of the red line for the highest temperatures measured in Figure S10a giving the activation energy of 866 ± 93 cm^–1^. The insets in Figures 3a,b show plots using eq 2 for the temperature dependence of emission intensity, and the value *E* = 1326 ± 75 cm^–1^ is obtained in Figure 3a, with a poorer fit to the lower data quality in Figure 3b. We therefore conclude that within the range of our measurements, spectral changes in lifetime and intensity involve an activation energy of ∼1000 ± 500 cm^–1^. This energy can be associated with the thermal occupation of the ^4^F_3/2_ level above ^4^G_5/2_. The thermal population of ^4^F_3/2_ reduces both the lifetime and the intensity of the spectrum. No new hot band spectral features are observed within the temperature range studied.

**Figure 3:**
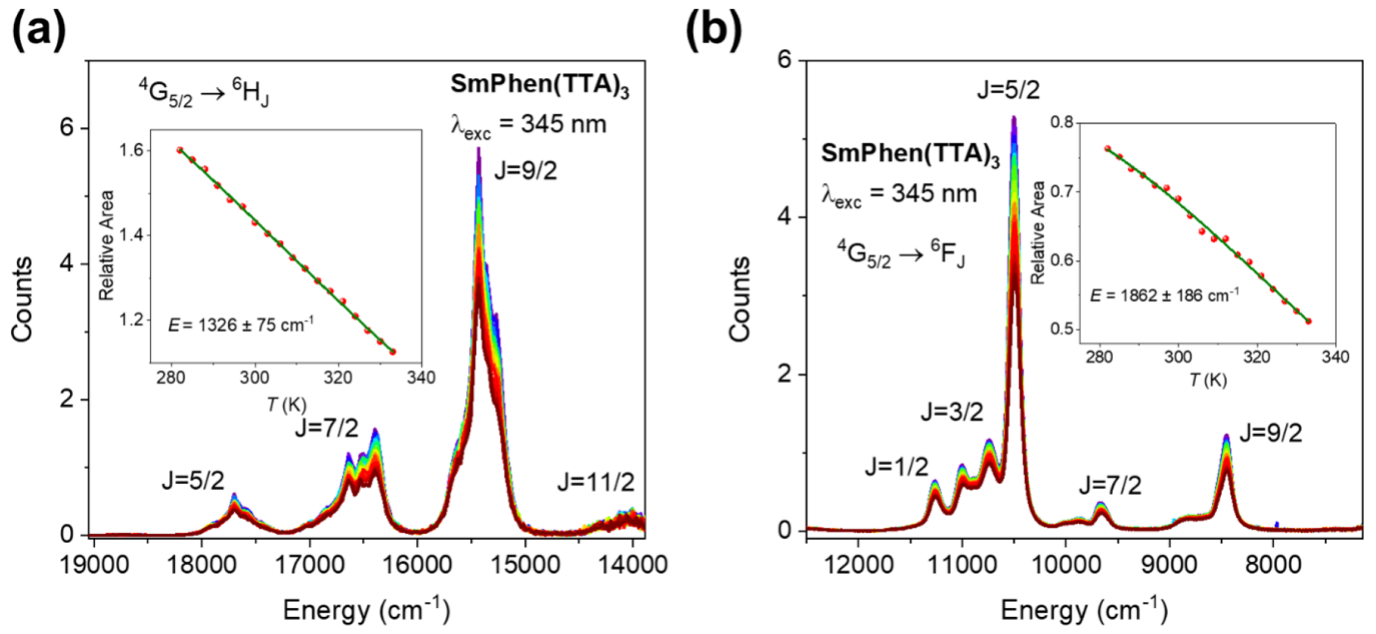
(a) Visible and (b) NIR emission spectra of 10 μM **SmPhen(TTA)**_**3**_ in toluene as a function of temperature after a Jacobian correction to energy scale. The insets show the integrated area of ^4^G_5/2_ → ^6^H_9/2_ (visible) and ^4^G_5/2_ → ^6^F_5/2_ (NIR) Sm^3+^ emission with temperature. The fits using eq 2 are displayed in olive. Purple: lowest temperature; red: highest temperature, as in Figure 2.

Just as for **SmPhen(TTA)**_**3**_, the thermal quenching of the luminescence intensity of **NdPhen(TTA)**_**3**_ is not as remarkable as that in **EuPhen(TTA)**_**3**_. The emission occurs from the two Kramers doublets of the ^4^F_3/2_ level at ∼11 400 cm^–1^, which is ∼5100 cm^–1^ above the next-lowest term ^4^I_15/2_ and ∼960 cm^–1^ below ^4^F_5/2_.^32,33^ The emission spectra between 282 and 333 K are shown in Figure 4a. Three red arrows mark the positions of hot band emission. In the range from 282 to 333 K, the emission intensity decreases by a factor of 1.1, whereas the lifetime increases by the same factor. The activation found from eq 2 for the decrease in the ^4^F_3/2_ → ^4^I_11/2_ peak area of **NdPhen(TTA)**_**3**_ with temperature in Figure 4a is found to be 693 ± 502 cm^–1^. The lifetime of the ^4^F_3/2_ state in **NdPhen(TTA)**_**3**_ is less than 1 μs and increases slightly with increasing temperature. The lifetime is much shorter than, for example, Y_3_Al_5_O_12_:Nd^3+^ (1 at. %), where the radiative and measured lifetimes are 250 and 228 μs, respectively,^34^ because the 5100 cm^–1^ gap to ^4^I_15/2_ can be spanned by three ∼1600 cm^–1^ vibrations in the present case. The decrease in emission intensity and yet increase in lifetime is intriguing, because they follow the same trend in Figure S11a but in fact work in opposite directions (Figure S11b). The excitation wavelength is similar in these experiments: 349 nm for the intensity measurement and 355 nm for the lifetime measurement. The difference between these lifetime and intensity plots shows an energy loss prior to entering the ^4^F_3/2_ state, presumably during transfer from the ligand. The state above ^4^F_3/2_ (i.e., ^4^F_5/2_) has a greater oscillator strength for the emission transition to the electronic ground state and a shorter luminescence lifetime than ^4^F_3/2_ (for example, see refs (35) and (36)). In fact, the decrease in ^4^F_3/2_ lifetime with decreasing temperature has been found by other authors, particularly in crystals with a higher concentration of Nd^3+^, and attributions to cross-relaxation, self-absorption, and multisite effects have been discounted as the major reason by Turri et al.^37^ We consider that the change in lifetime with temperature could result from (i) thermalization within the two Kramers doublets of ^4^F_3/2_ and/or (ii) back-energy transfer to Nd^3+4^F_3/2_ from a trap, such as Yb^3+^ present in the sample. The lifetime of the ^2^F_5/2_ state of Yb^3+^ in **YbPhen(TTA)**_**3**_ (see below) is longer than that of the ^4^F_3/2_ Nd^3+^ state in **NdPhen(TTA)**_**3**_, and it is situated at ∼1150 cm^–1^ to lower energy (i.e., −1150 cm^–1^). The three-parameter single-barrier fit eq 1 to the lifetime data in Figure 4b in the inset gives the values *k*_r_ = (9.6 ± 0.1) × 10^5^ s^–1^, Γ_0_ = 22 ± 34 s^–1^, and *E* = −1699 ± 293 cm^–1^. However, the adjusted coefficient of determination, *R*_adj_^2^ = 0.99072 and the value of *k*_r_ would infer a quantum yield around 90%. Restraining the quantum yield to less than 50% and using separate fits to the two fairly linear portions of the curve gives much smaller values of *E* around −150 cm^–1^.

**Figure 4:**
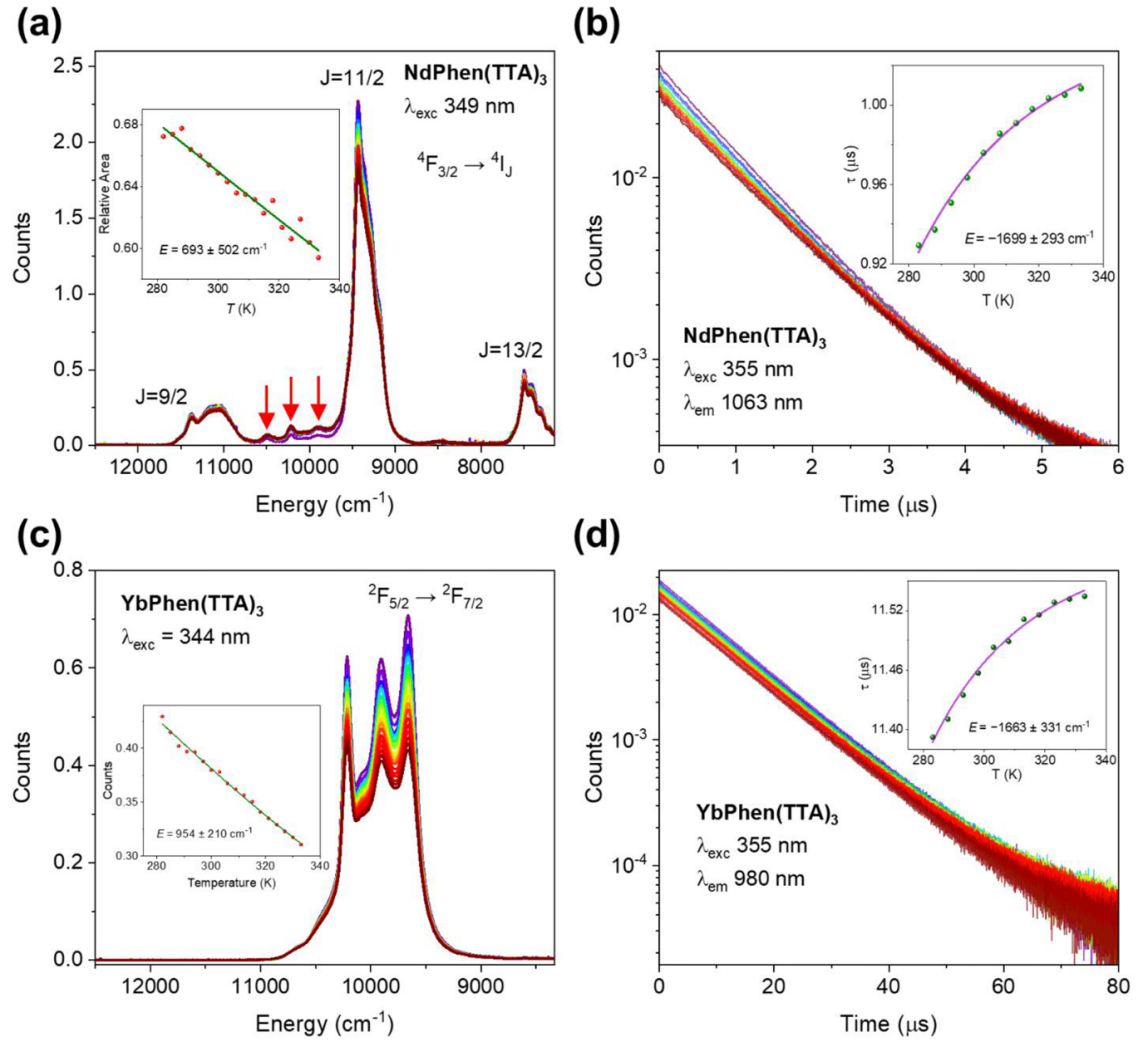
Emission spectra of 10 μM (a) **NdPhen(TTA)**_**3**_ and (c) **YbPhen(TTA)**_**3**_ in toluene as a function of temperature after a Jacobian correction to energy scale. The insets show the integrated areas of the emission transitions ^4^F_3/2_ → ^4^I_11/2_ Nd^3+^ and ^2^F_5/2_ → ^2^F_7/2_ Yb^3+^, respectively, with temperature with fits using eq 2. The decay curves of 10 μM (b) **NdPhen(TTA)**_**3**_ and (d) **YbPhen(TTA)**_**3**_ emission in toluene with varying temperature. The insets show the variation in lifetime with temperature with fits by eq 1. Purple: lowest temperature; red: highest temperature, as in Figure 2.

The 4f^13^ system Yb^3+^ comprises only two ^2*S*+1^*L*_*J*_ multiplet terms: the ground state term ^2^F_7/2_ (comprising four Kramers doublets) and the excited ^2^F_5/2_ term at ∼10 200 cm^–1^ (comprising three Kramers doublets) (Figures S1 and S12a). The temperature variation of the **YbPhen(TTA)**_**3**_ emission spectrum and lifetime are displayed in Figure 4c,d. The spectrum has previously been reported at slightly higher resolution, with an estimated quantum yield of 0.16%.^38^ The lifetime does not show a significant change when the concentration in toluene is increased by a factor of 10 (Figure S12b) showing that solvent quenching effects may not be very important. Although the trends of intensity and lifetime are similar in Figure S12c, over the temperature range studied, the lifetime only increases by a factor of 1.01, whereas the intensity decreases by a factor of 1.38 (Figure S12d). The single-barrier quenching model, eq 2, gives the activation energy for Figure 4c of 954 ± 210 cm^–1^, with the fit shown in the inset. Similar to the trap in **NdPhen(TTA)**_**3**_, this is in reasonable agreement with the separation of ∼1150 cm^–1^ from the ^4^F_3/2_ level of Nd^3+^. However, the fit of lifetime data in Figure 4d using eq 1 gives the activation energy of −1663 ± 331 cm^–1^, with a quantum yield of nearly 100%. Using the above quantum yield to estimate *k*_r_ in an Arrhenius equation gives a poor fit (*R*_adj_^2^ = 0.955) with *E* = −17 cm^–1^.

In conclusion, we have investigated the occurrence of thermally activated processes occurring near room temperature for **LnPhen(TTA)**_**3**_ complexes. Dominant back-transfer from Eu^3+^ to a charge-transfer state has been demonstrated for Ln = Eu, analogous to the solid-state study of Berry et al.^26^ The derived energies from the back-transfer model for Ln = Sm, Nd, and Yb cannot be linked to the ligand or charge transfer states. Ln = Sm^3+^ exhibits quenching of intensity and lifetime with increasing temperature, and this has been associated with thermal occupation of higher levels. By contrast, Ln = Nd, Yb exhibit intensity quenching and lifetime lengthening with increasing temperature. The two effects cannot be consistently modeled using the common back-transfer models. We infer that the intensity decrease with increasing temperature is related to the population mechanism from the ligand, whereas the lifetime change with temperature subsequently occurs in the lanthanide excited state. A detailed investigation of the ligand–lanthanide energy transfer is therefore required for these cases.

## Supporting Information Available

Syntheses; instruments; calculations; energy level diagrams; FT-IR spectra; absorption spectra of LnPhen(TTA)_3_ in toluene; trends in intensity and lifetime for EuPhen(TTA)_3_; fitting of EuPhen(TTA)_3_ lifetime and intensity data vs temperature; emission spectra of LnPhen(TTA)_3_ in 10 μM toluene; Arrhenius plots of Eu(thd)_3_ and EuPhen(TTA)_3_; absorption spectra of LnPhen(TTA)_3_ (Ln = Gd, Eu) in 1 mM toluene solution; Arrhenius plots of the nonradiative decay rate of the ^4^G_5/2_ level of SmPhen(TTA)_3_; plots of functions of lifetime and integrated intensity data as a function of temperature and inverse temperature for NdPhen(TTA)_3_; 77 K emission spectrum of YbPhen(TTA)_3_; luminescence decay of YbPhen(TTA)_3_ in toluene; spectral data plots of YbPhen(TTA)_3_ versus temperature and inverse temperature (PDF)
Transparent Peer Review report available (PDF)
