# Supplementary material for: Thermally Activated Photophysical Processes of Organolanthanide Complexes in Solution
Source: J Phys Chem Lett. 2022 May 26;13(21):4800–6. doi: 10.1021/acs.jpclett.2c01350 (PMC9169037; doi:10.1021/acs.jpclett.2c01350)
Supplement: Supplementary file 1 — jz2c01350_si_001.pdf [file jz2c01350_si_001.pdf]

SUPPORTING INFORMATION

Thermally Activated Photophysical Processes of Organo-Lanthanide Complexes in Solution

Waygen Thor,[a] Hei-Yui Kai,[a] Yonghong Zhang,[a,b] Ka-Leung Wong,[a*] Peter A. Tanner[a*]

[a]Department of Chemistry, Hong Kong Baptist University, Waterloo Road, Kowloon Tong, Hong Kong S.A.R. P. R. China

[b]State Key Laboratory of Chemistry and Utilization of Carbon Based Energy Resources, Key Laboratory of Oil and Gas Fine Chemicals, Ministry of Education & Xinjiang Uygur Autonomous Region, Urumqi Key Laboratory of Green Catalysis and Synthesis Technology, College of Chemistry, Xinjiang University, Urumqi 830046, Xinjiang, P. R. China.

K.-L.W. E-mail: klwong@hkbu.edu.hk

P.A.T. E-mail: peter.a.tanner@gmail.com

Table of Contents

		Page
A	Materials	S2
B	Syntheses of Complexes	S3
C	Instruments	S3
D	Calculations	S3
Figure S1	Energy level diagrams for the lanthanide ions studied with representative energy levels labeled	S4
Figure S2	The experimental and calculated FTIR spectra of $SmPhen(TTA)_3$	S4
Figure S3	(a) The absorption spectra of $LnPhen(TTA)_3$ in 10 μM toluene at room temperature. (b) The absorption peak maxima with respect to the ionic radii of Ln^{3+} ion	S5
Figure S4	The absorption spectra of $LnPhen(TTA)_3$ in 10 μM toluene with temperature for Ln = (a) La, (b) Nd, (c) Eu, (d) Gd and (e) Yb	S6
Figure S5	(a) Trends in intensity and lifetime; (b) Arrhenius-type plots of lifetime and emission intensity for $EuPhen(TTA)_3$ at different temperatures.	S7
E	Back Energy Transfer to Two States	
Figure S6	Fitting of $EuPhen(TTA)_3$ lifetime and intensity data vs temperature by two barrier model.	S8
Figure S7	The emission spectra of $LnPhen(TTA)_3$ in 10 μM toluene where Ln is (a) Y (b) La (c) Sm (d) Eu (e) Gd at 77 K	S9
Figure S8	Arrhenius plots: (a) Nonradiative decay rate of (a) europium tris(2,2,6,6-tetramethyl-3,5-heptanedionato) (with data from Berry et al.), (b) $EuPhen(TTA)_3$, with inverse temperature	S10
Figure S9	(a) The absorption spectra of $LnPhen(TTA)_3$ (Ln = Gd, Eu) in 1 mM toluene solution at room temperature. (b) The subtraction of $EuPhen(TTA)_3$ spectrum from $GdPhen(TTA)_3$	S10
Figure S10	Arrhenius plots of nonradiative decay rate of the $^4G_{5/2}$ level of $SmPhen(TTA)_3$ from measurements of (a) $^4G_{5/2} \rightarrow {}^6H_{9/2}$ visible and (b) $^4G_{5/2} \rightarrow {}^6F_{5/2}$ near infrared lifetimes	S11
Figure 11	Plots of functions of lifetime and integrated intensity data as a function of temperature and inverse temperature for $NdPhen(TTA)_3$	S11
Figure S12	(a) 77 K emission spectrum of $YbPhen(TTA)_3$. The locations of the $^2F_{5/2}$ luminescent state and $^2F_{7/2}$ ground state levels are marked. (b) The luminescence decay of $YbPhen(TTA)_3$ at 100 μm concentration in toluene at different temperatures. (c), (d) Spectral data plots of $YbPhen(TTA)_3$ versus temperature and inverse temperature	S12
References		S12

A. Materials

$LnCl_3.6H_2O$ and $Eu(NO_3)_3.6H_2O$ were purchased from Sigma-Aldrich or TCI (all ≥99.99% trace metals basis) and used without further purification. All organic chemicals were purchased from TCI or Energy Chemical with 98% purity and used without further purification.

B. Syntheses of complexes

Synthesis of LnPhen(TTA)$_3$ complexes

The **EuPhen(TTA)$_3$** samples were synthesized by the literature method.[1] Typically, TTA (3 mmol) and phen (1 mmol) were dissolved in 15 mL ethanol in a flask, with stirring at room temperature. Then, the pH of the solution was adjusted to 7.0 by the addition of NaOH solution (1.0 M). After that, 1 mmol of $EuCl_3$ solution in 5.0 mL of deionized water were then added into the above mixture at 60 ^oC with vigorous stirring for 1.0 h to ensure a complete precipitation. The precipitate was finally filtered, washed repeatedly with ethanol and water, and dried overnight under vacuum.

EuPhen(TTA)$_3$: Yield: 57%; Elemental analysis: found (%): C 46.27; H 2.25; N 2.82. calcd. (%): C 43.43; H 2.02; N 2.81. HRMS (ESI) calcd for $C_{24}H_{12}EuF_9NaO_6S_3^+$ $[Eu(TTA)_3+Na]^+$ 838.8757, found 838.8754. HRMS (ESI) calcd for $C_{25}H_{13}EuF_9NaO_8S_3^+$ $[Eu(TTA)_3+COOH]^-$ 860.8847, found 860.8826.

C. Instrumental

The ultraviolet-visible absorption spectra were measured in solution in the range 200-800 nm by a Perkin Elmer® LAMBDA 1050+ UV/VIS/NIR double beam spectrophotometer. The emission, excitation spectra and luminescence decay curves in the visible region were recorded using a Horiba Fluorolog®-3 instrument with a 450 W xenon lamp. The signal was detected by a Hamamatsu R928 photomultiplier and corrected with excitation and emission correction factors to eliminate response characteristics. The luminescence decay curves in the NIR region were measured by a digital phosphor oscilloscope (1 GHz, 20 GS s^{-1}) with the Nd:YAG pulsed laser as the excitation source. The laser system consisted of a Nd:YAG pump laser, a third-order harmonic generator (THG at 355 nm, 120 mJ), and an optical parametric oscillator (OPO, Spectra-Physics versaScan and UVScan) with a pulse duration of 8 ns and repetition frequency of 10 Hz. A custom-made liquid nitrogen cryostat with a NMR tube sample holder was employed for 77 K studies. A qX3/Horiba4 variable temperature cell was employed to investigate the temperature dependence of emission between 280-323 K.

D. Calculations

Calculations were performed using Orca version 4.2.1[2-3] and accessories, together with the use of Avogadro.[4] Various functionals as listed in Table S1 were employed with basis sets def2-TZVP with the Stuttgart in-core effective core potentials[5] and basis sets for the lanthanide ions.[6-7] In all optimizations the Grimme dispersion correction was used in the calculations.[8-9] In ORCA, the integration grid was usually set to 6 (Lebedev 590 points) with the final grid 7 (Lebedev 770 points). The nature of the optimized structures was checked with frequency calculations. The

simulated absorption spectra employed the bandwidth of 3000 cm^{-1}. Solvent effects were taken into account by the Conductor-like Polarizable Continuum Model (CPCM) model[10-11] in ORCA.

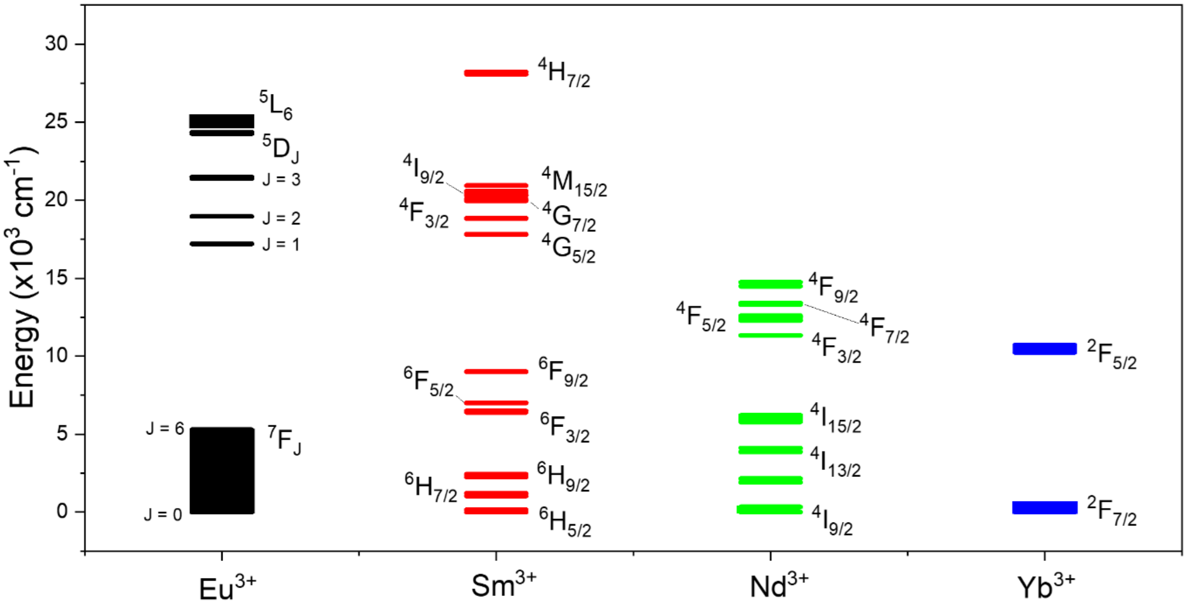

Figure S1. Energy level diagrams for the lanthanide ions studied with representative energy levels labeled.

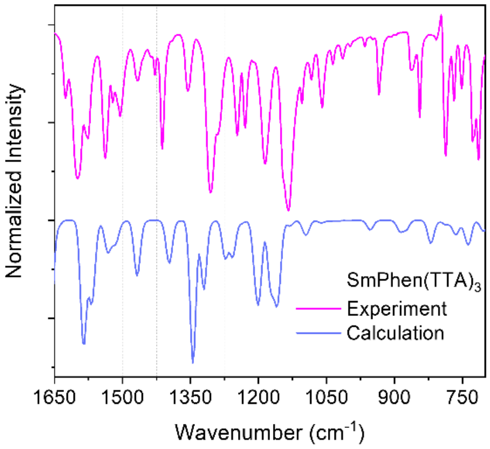

Figure S2. The experimental and calculated FTIR spectra of **SmPhen(TTA)3**. The frequency calculation was performed at PBE0/def2-TZVP level of theory.

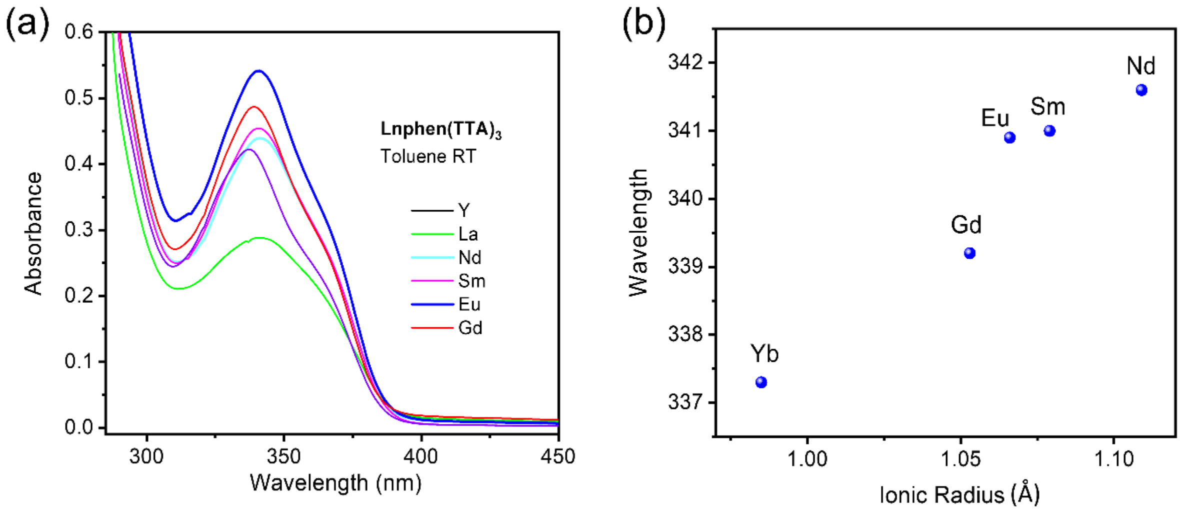

Figure S3. (a) The absorption spectra of **LnPhen(TTA)₃** in 10 µM toluene at room temperature. (b) The absorption peak maxima with respect to the ionic radii of Ln^{3+} ion. A general red-shifting in the absorption maxima can be observed with increasing ionic radii.

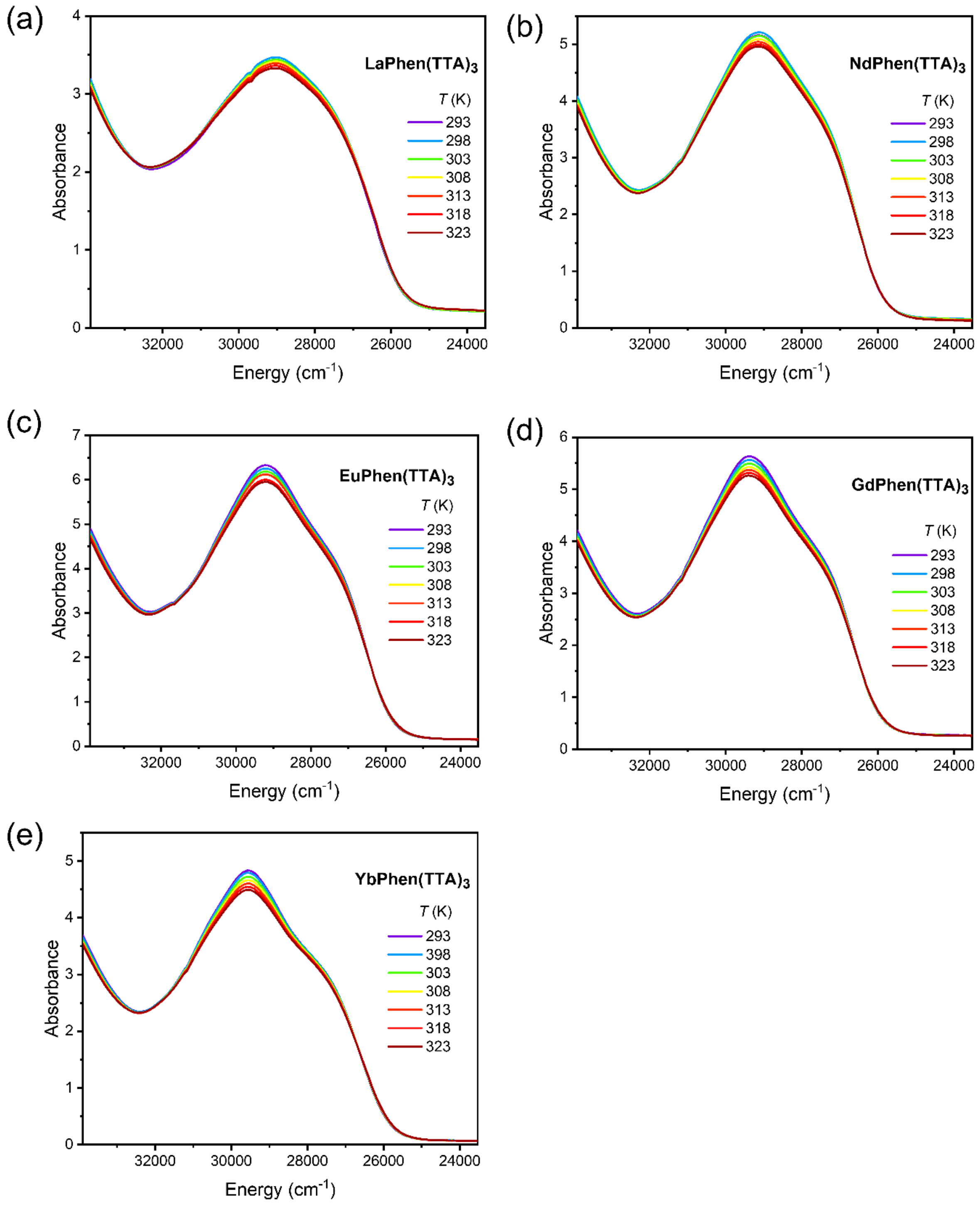

Figure S4. The absorption spectra of **LnPhen(TTA)$_3$** in 10 μM toluene with temperature for Ln = (a) La, (b) Nd, (c) Eu, (d) Gd and (e) Yb. The absorption spectra have been corrected with the thermal expansion of toluene (0.00108 mL K^{-1}).

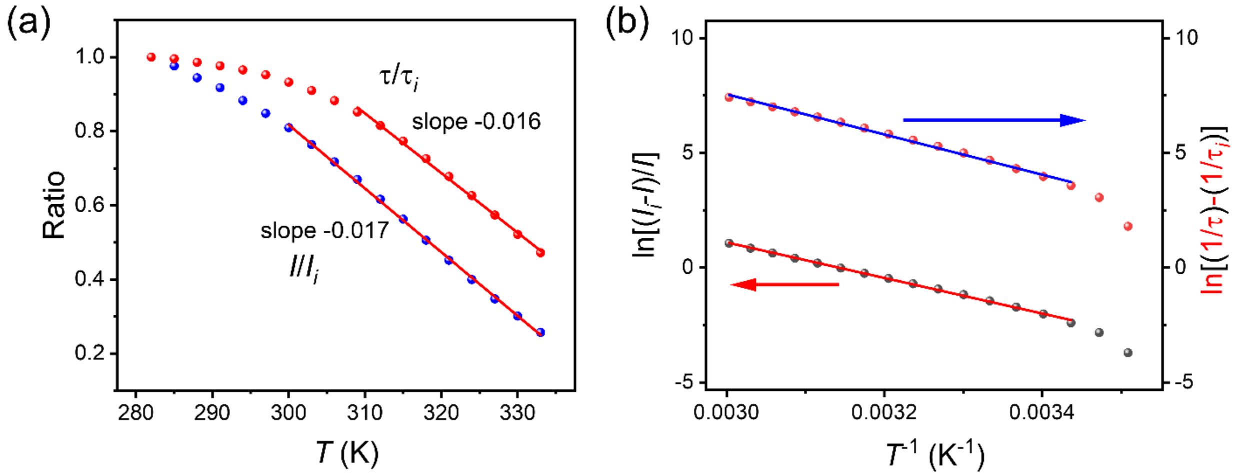

Figure S5. (a) Trends in intensity and lifetime; (b) Arrhenius-type plots of lifetime and emission intensity for **EuPhen(TTA)3** at different temperatures. Here, the subscript *i* refers to the initial data point. The plots show the same trend after about 305 K. The equations employed are from[12].

E. Back Energy Transfer to Two States

To describe the back energy transfer (BET) properties with the inclusion of the BET from $^5D_0 \rightarrow {}^5D_1$, Eq. 1 and 2 can be modified and described as:

$$\tau(T) = \frac{1}{\mathrm{A}+B\exp\left(-\frac{E}{kT}\right)+\mathrm{C}\exp\left(-\frac{E_{5D1}}{kT}\right)} \quad \text{(S1)}$$

$$\frac{I(T)}{I_0} \approx \frac{1}{1+A\exp\left(-\frac{E}{kT}\right)+\mathrm{B}\exp\left(-\frac{E_{5D1}}{kT}\right)} \quad \text{(S2)}$$

where E_{5D1} is the energy gap between $^5D_0$ and $^5D_1$ (1746 cm^{-1}). However, the fitted graphs and parameters are not as good as using Eqs. (1) and (2). This indicates that in the case of Eu^{3+}, the thermal quenching process is dominated by only single process where the back energy transfer from $^5D_0$ to $^5D_1$ is not considered.

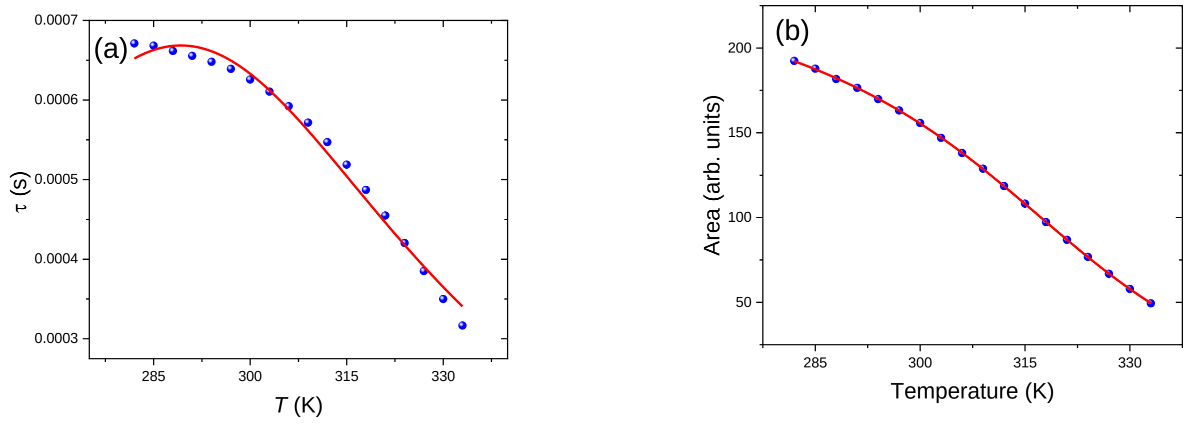

Figure S6. Fitting of **EuPhen(TTA)$_3$** lifetime and intensity data vs temperature by two barrier model.

The fit to (a) with 4 free parameters is:

$$\tau(T) = \frac{1}{(-4.2\text{E}3 \pm 1.3\text{E}4) + (7.4E6 \pm 2.0E6)\exp\left(-\frac{(1746 \pm 0)}{kT}\right) + (3.9E2 \pm 3.3E3)\exp\left(-\frac{(-499 \pm 1042)}{kT}\right)}$$

where k = 0.6950536 cm^{-1} K^{-1}, with R_{adj}^2 = 0.98699.

The fit to (b) with 4 free parameters is:

$$\frac{I(T)}{241 \pm 4} \approx \frac{1}{1 + (1703 \pm 210)\exp\left(-\frac{(1746 \pm 0)}{kT}\right) + (1.2E12 \pm 6.0E12)\exp\left(-\frac{(6178 \pm 1177)}{kT}\right)}$$

where k = 0.6950536 cm^{-1} K^{-1}. Although R_{adj}^2 = 0.99997 the parameter 1.2E12±6.0E12 is not meaningful.

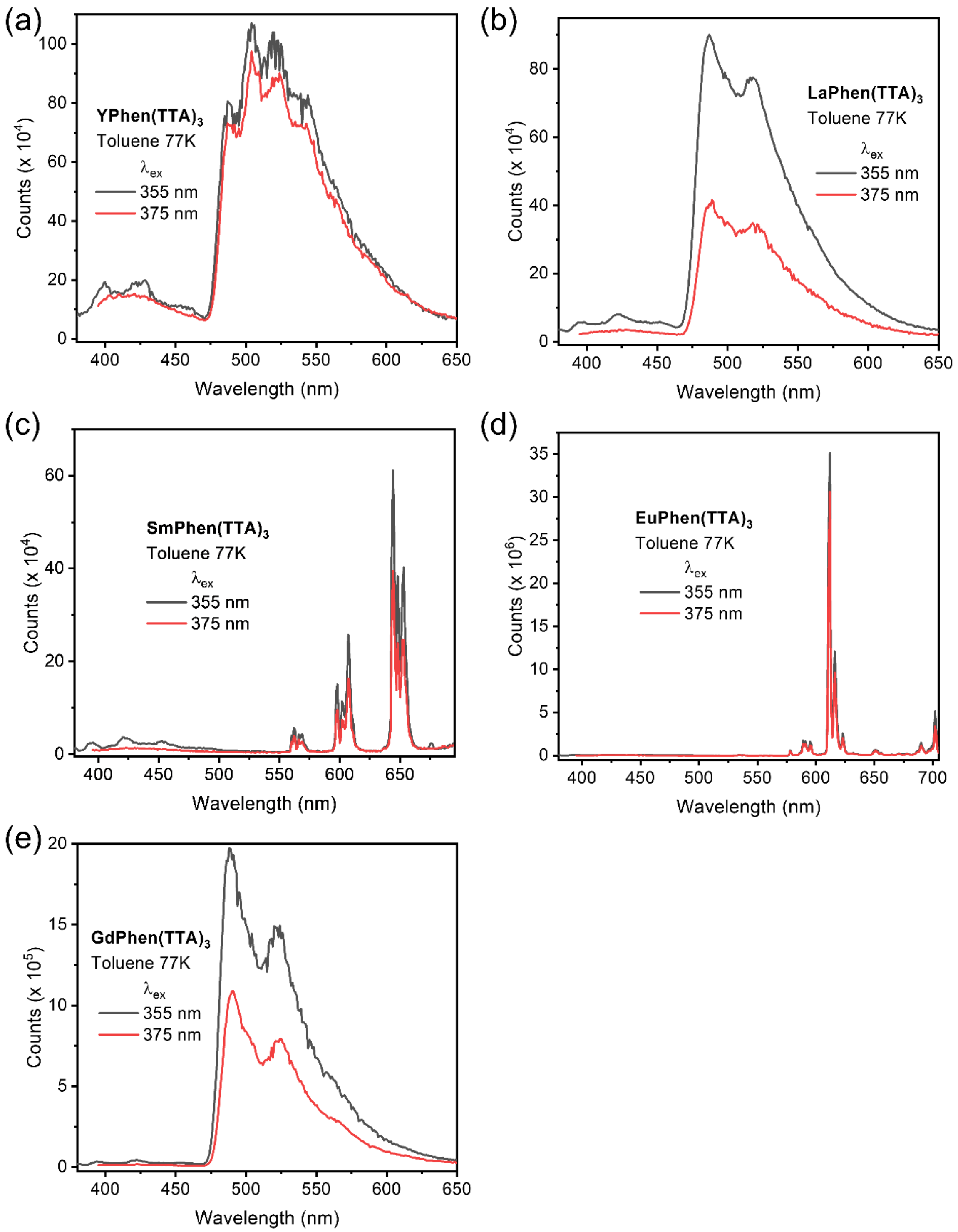

Figure S7. The emission spectra of **LnPhen(TTA)₃** in 10 µM toluene where Ln is (a) Y (b) La (c) Sm (d) Eu (e) Gd at 77 K. A triplet state located at ~488 nm can be observed for Ln = Y, La, Gd and Lu.

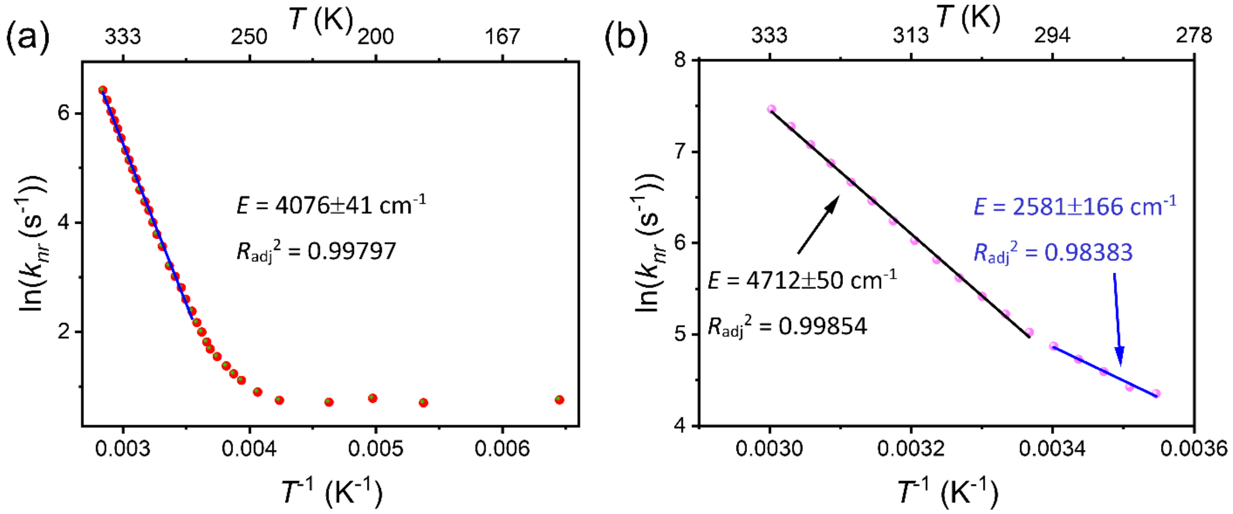

Figure S8. Arrhenius plots: (a) Nonradiative decay rate of (a) europium tris(2,2,6,6-tetramethyl-3,5-heptanedionato) (with data from Berry et al.[13]), (b) **EuPhen(TTA)$_3$**, with inverse temperature. The Arrhenius equation employed is $\ln(k_{nr}) = A - E/kT$ where k is the Boltzmann constant.

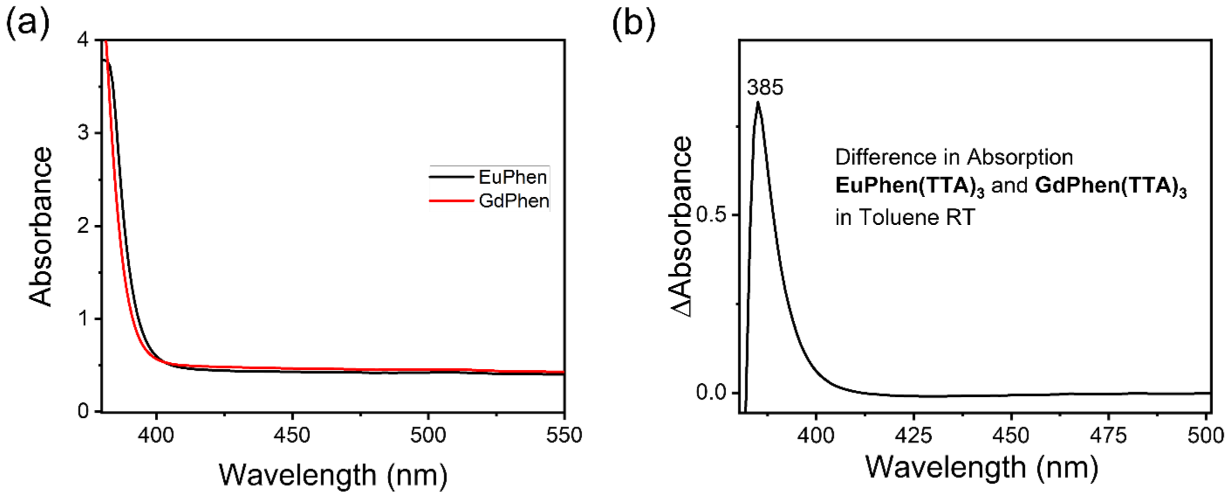

Figure S9. (a) The absorption spectra of **LnPhen(TTA)$_3$** (Ln = Gd, Eu) in 1 mM toluene solution at room temperature. (b) The subtraction of **EuPhen(TTA)$_3$** spectrum from **GdPhen(TTA)$_3$** showing a band at *ca*. 385 nm, indicating the possible location of the vertical transition of the charge transfer state.

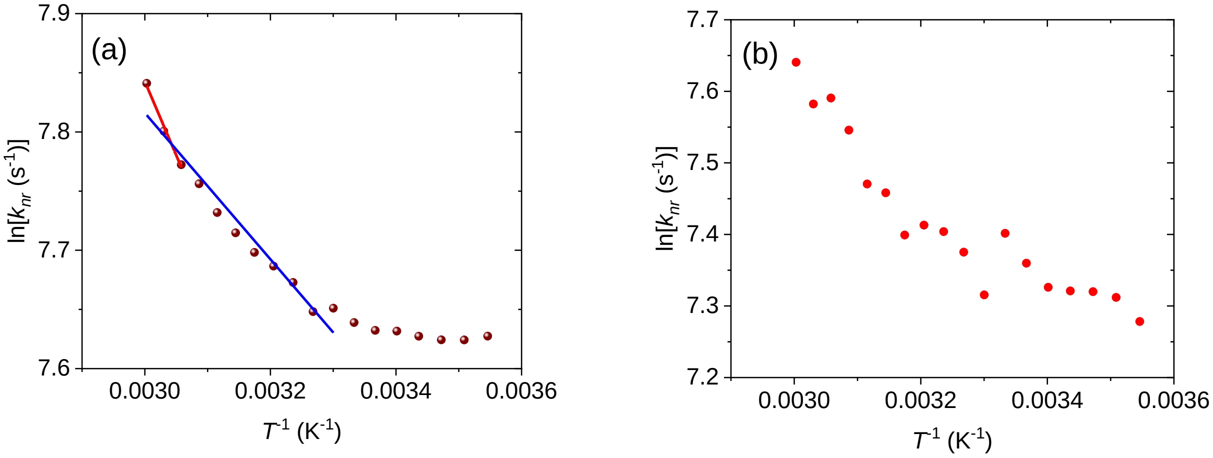

Figure S10. Arrhenius plots of nonradiative decay rate of the $^4G_{5/2}$ level of **SmPhen(TTA)₃** from measurements of (a) $^4G_{5/2} \rightarrow {}^6H_{9/2}$ visible and (b) $^4G_{5/2} \rightarrow {}^6F_{5/2}$ near infrared lifetimes. The radiative decay rate of $^4G_{5/2}$ was estimated from the 77 K value of measurement of 93.8 μs for **SmPhen(TTA)₃**. The red and blue slopes in (a) give values of activation energy of 866 cm^{-1} and 430 cm^{-1}, respectively.

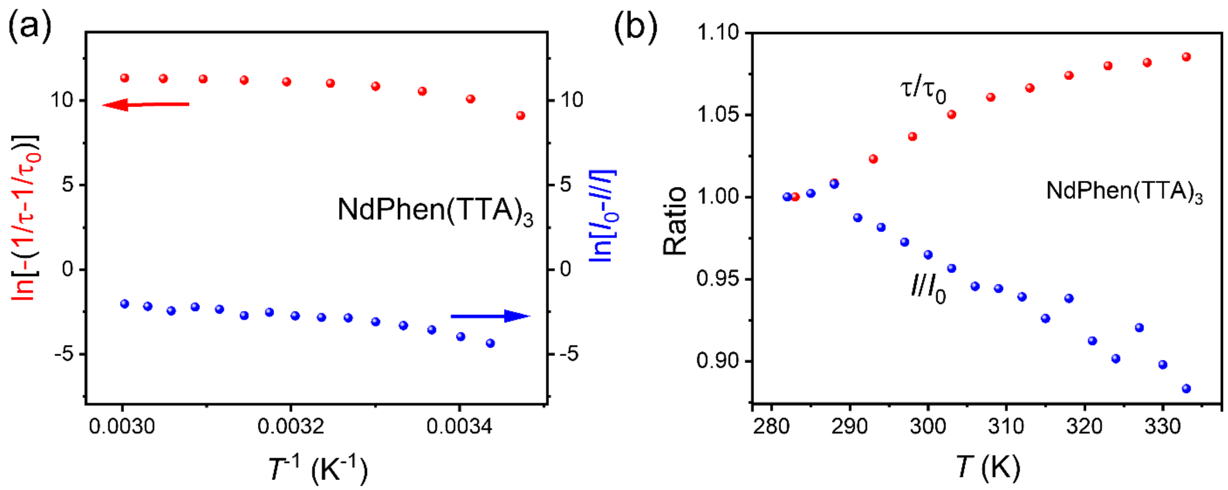

Figure S11. Plots of functions of lifetime and integrated intensity data as a function of temperature and inverse temperature for **NdPhen(TTA)₃**.

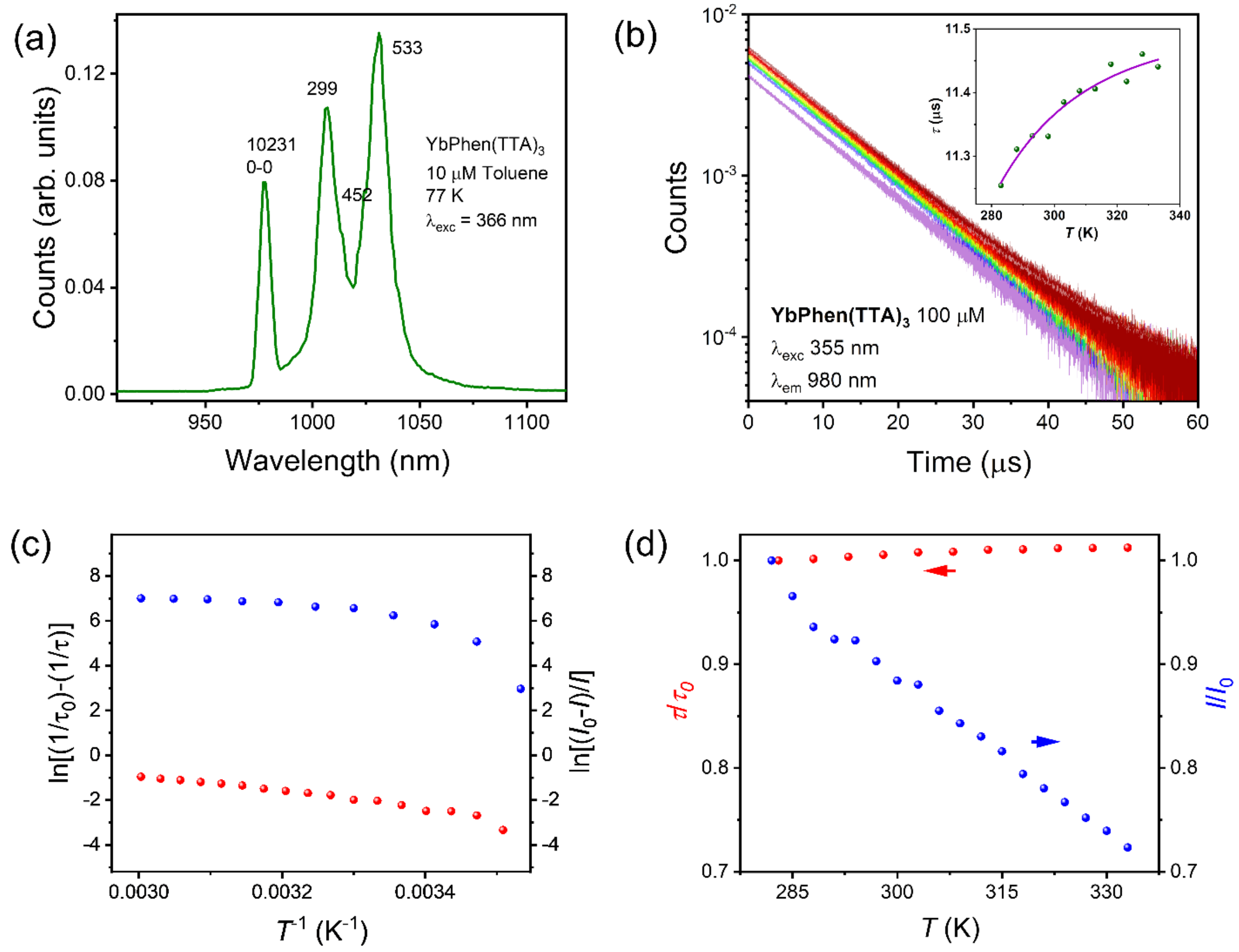

Figure S12. (a) 77 K emission spectrum of **YbPhen(TTA)3**. The locations of the $^2F_{5/2}$ luminescent state and $^2F_{7/2}$ ground state levels are marked. (b) The luminescence decay of **YbPhen(TTA)3** at 100 μm concentration in toluene at different temperatures. The inset shows the calculated lifetime of Yb^{3+} versus temperature, with the fit using Eq. (1) displayed in purple. Purple: lowest temperature; Red: highest temperature. (c), (d) Spectral data plots of **YbPhen(TTA)3** versus temperature and inverse temperature.

References

(1) Meng, M.; Bai, M.; Da, Z.; Cui, Y.; Li, B.; Pan, J., Selective Recognition of Salicylic Acid Employing New Fluorescent Imprinted Membrane Functionalized with Poly(Amidoamine) (PAMAM)-Encapsulated Eu(TTA)3phen. *J. Lumin.* **2019**, *208*, 24-32.

(2) Neese, F., The Orca Program System. *WIREs Comput Mol Sci.* **2012**, *2*, 73-78.

(3) Neese, F., Software Update: The Orca Program System, Version 4.0. *WIREs Comput Mol Sci.* **2018**, *8*, e1327.

(4) Hanwell, M. D.; Curtis, D. E.; Lonie, D. C.; Vandermeersch, T.; Zurek, E.; Hutchison, G. R., Avogadro: An Advanced Semantic Chemical Editor, Visualization, and Analysis Platform. *J. Cheminformatics* **2012**, *4*, 17.

(5) Energy-Consistent Pseudopotentials of the Stuttgart/Cologne Group. http://www.tc.uni-koeln.de/PP/clickpse.en.html.

(6) Andrae, D.; Häußermann, U.; Dolg, M.; Stoll, H.; Preuß, H., Energy-Adjustedab Initio Pseudopotentials for the Second and Third Row Transition Elements. *Theor. Chim. Acta* **1990**, *77*, 123-141.

(7) Dolg, M.; Stoll, H.; Savin, A.; Preuss, H., Energy-Adjusted Pseudopotentials for the Rare Earth Elements. *Theor. Chim. Acta* **1989**, *75*, 173-194.

(8) Grimme, S.; Ehrlich, S.; Goerigk, L., Effect of the Damping Function in Dispersion Corrected Density Functional Theory. *J. Comput. Chem.* **2011**, *32*, 1456-1465.

(9) Grimme, S.; Antony, J.; Ehrlich, S.; Krieg, H., A Consistent and Accurate Ab Initio Parametrization of Density Functional Dispersion Correction (Dft-D) for the 94 Elements H-Pu. *J. Chem. Phys* **2010**, *132*, 154104.

(10) Tomasi, J.; Mennucci, B.; Cammi, R., Quantum Mechanical Continuum Solvation Models. *Chem. Rev.* **2005**, *105*, 2999-3094.

(11) Barone, V.; Cossi, M., Quantum Calculation of Molecular Energies and Energy Gradients in Solution by a Conductor Solvent Model. *J. Phys. Chem. A* **1998**, *102*, 1995-2001.

(12) Blasse, G.; Bril, A.; de Poorter, J. A. Radiationless Transitions in the Eu^{3+} Center in $LaAlO_3$. J. Chem. Phys. **1970**, 53, 4450-4453.

(13) Berry, M. T.; Stanley May, P.; Xu, H. Temperature Dependence of the Eu^{3+} $^5D_0$ Lifetime in Europium Tris(2,2,6,6-tetramethyl-3,5-heptanedionato). *J. Phys. Chem.* **1996**, *100*, 9216-9222.
