# Supplementary material for: Thermally Activated Photophysical Processes of Organolanthanide Complexes in Solution
Source: J Phys Chem Lett. 2022 May 26;13(21):4800–6. doi: 10.1021/acs.jpclett.2c01350 (PMC9169037; doi:10.1021/acs.jpclett.2c01350)
Supplement: Supplementary file 2 — jz2c01350_si_002.pdf [file jz2c01350_si_002.pdf]

jz-2022-01350n.R1

Name: Peer Review Information for "Thermally Activated Photophysical Processes of Organo-Lanthanide Complexes in Solution"

First Round of Reviewer Comments

Reviewer: 1

Comments to the Author

In this work, Thor et. al. presented a nice work on the activation energy of organo-lanthanide compound with Ln = Eu, Sm, Nd and Yb. By using spectroscopic method, they investigated the thermal effect on the emission intensity and the luminescence lifetime. They demonstrated that the assumption of t/ t0 = I/I0 does not hold. Besides concluding that the thermal quenching for both Ln = Eu and Sm are due to higher receiving states, the increasing lifetime with increasing temperature of Nd and Yb are intriguing, which require the attention from the community. I support the publication of this work after the authors addressed some of these questions:

1. How is the charge transfer state energy related to ratio of ED/MD of Eu3+? The authors should include the reference for readers.

2. Is it possible to observe any CT band from the emission spectra in cold correspond to the activation energy found, e.g., in the Eu-complex?

3. Although it seems that the author discussed the non-radiative rate of Sm3+ with temperature, but it might be also good to discuss the activation energy obtained for the radiative rate of Sm3+ in the NIR region.

4. The authors discussed the two states back energy transfer in the main text and supplementary information, but it is nice also to include the fitting graph to demonstrate that the equation is not well fitted.

Reviewer: 2

Comments to the Author

This manuscript entitled: “Thermally Activated Photophysical Processes of OrganoLanthanide Complexes in Solution” focuses on the mechanisms of thermal quenching properties for different Ln3+ ions of complex LnPhen(TTA)3. They look deep into the energies from the back-transfer model for Ln = Sm, Nd and Yb cannot be linked to the ligand or charge transfer states. When Ln = Sm3+ exhibits quenching of intensity and lifetime with increasing temperature. On the other hand, Ln = Nd, Yb exhibit intensity quenching and lifetime lengthening with increasing temperature. This work is quite interesting, but it

remains something that needs to be explained and discussed more clearly. We suggest that this paper be minor revised before publishing in The Journal of Physical Chemistry Letters.

1. In this article, the authors reported the thermally activated photoluminescence properties of organo-lanthanide complexes by using different lanthanide ions. However, the readers may lose focus because of the various effect of temperature analysis descriptions.

2. Energy level diagrams for the lanthanide ions have been marked in figure S1. However, some energy levels do not appear in this figure which may confuse the readers. Please mark all the energy levels clearly.

Author's Response to Peer Review Comments:

Response to Reviewer's Comments

We are grateful to the Reviewers for reading the manuscript carefully and for the constructive comments given. These have now been taken into account in the revised version of the manuscript as detailed below.

Reviewer: 1

Recommendation: This paper is publishable subject to minor revisions noted. Further review is not needed.

Comments:

In this work, Thor *et. al.* presented a nice work on the activation energy of organo-lanthanide compound with Ln = Eu, Sm, Nd and Yb. By using spectroscopic method, they investigated the thermal effect on the emission intensity and the luminescence lifetime. They demonstrated that the assumption of $\tau/\tau_0 = I/I_0$ does not hold. Besides concluding that the thermal quenching for both Ln = Eu and Sm are due to higher receiving states, the increasing lifetime with increasing temperature of Nd and Yb are intriguing, which require the attention from the community. I support the publication of this work after the authors addressed some of these questions:

Comment 1: How is the charge transfer state energy related to ratio of ED/MD of Eu^{3+}? The authors should include the reference for readers.

Response: Thank you for your suggestion. We are unaware of studies that have compared the location of the CT state with the ED/MD ratio of Eu^{3+} emission. However, the reasoning is clear, except other factors may be involved, such as the nature of the ligand and metal-ligand bond length, etc. The CT transition is a strong ED allowed transition and its intensity can mix with $^5D_0 \rightarrow {}^7F_2$ by the odd components of the crystal field and promote greater intensity. The degree of mixing is inversely proportional to the energy separation of the CT band from the $^5D_0$ state. On the other hand, the MD intensity is fixed for $^5D_0 \rightarrow {}^7F_1$. We have included the following explanation in the manuscript:

Generally, although other parameters may be involved, the charge transfer energy can be related to the electric dipole : magnetic dipole ratio of the emission spectrum since the $^5D_0 \rightarrow {}^7F_2$ Eu^{3+} emission intensity can be enhanced whereas $^5D_0 \rightarrow {}^7F_1$ is not, to first order. The degree of enhancement is inversely proportional to the energy separation of the CT band from that of $^5D_0$. Taking the ratio $({}^5D_0 \rightarrow {}^7F_2)/({}^5D_0 \rightarrow {}^7F_1) = R$ as an illustration, the value of R for $Eu(thd)_3$ is 19.9 whereas it is 13.0 for **EuPhen(TTA)$_3$**. The charge transfer band is therefore expected at higher energy in the present case than for $Eu(thd)_3$.

Comment 2: Is it possible to observe any CT band from the emission spectra in cold correspond to the activation energy found, e.g., in the Eu-complex?

Response: As mentioned in the text, the activation energy refers to a crossover point of the CT parabola with the $4f^N$ manifold. This is distinct from the vertical excitation energy of the CT state. Hence the value of activation energy does not relate to the CT spectral band position. Also it is unlikely that CT emission would occur.

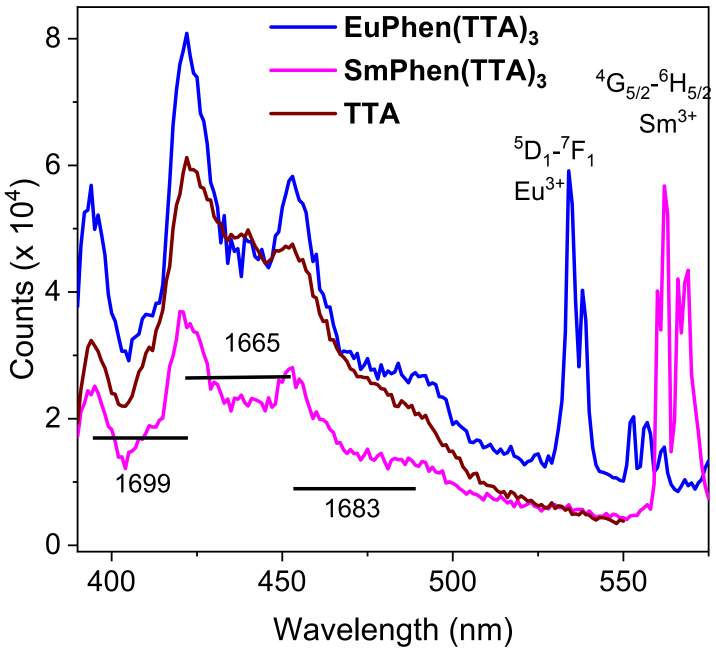

77 K spectrum of TTA ligand and complexes initially at 10 μM concentration in toluene.

The UV spectral region is dominated by the $S_1 – S_0$ ligand emission, with vibrational progression frequency 1682±17 cm^{-1} so that an underlying broad CT band would not be visible.

Comment 3: Although it seems that the author discussed the non-radiative rate of Sm^{3+} with temperature, but it might be also good to discuss the activation energy obtained for the radiative rate of Sm^{3+} in the NIR region.

Response: Thank you for the suggestion. We have included the activation energy deduced from visible emission in Figure S10a. The plot for NIR emission (Figure S10b) is rather scattered due

to poorer data quality, but of the same trend, so we have not fitted it. We included/modified the following sentences in the manuscript.

Abstract: The emission intensity and lifetime were also quenched with increasing temperature for Ln = Sm and the activation energy for nonradiative decay is similar to that for the thermal population of Sm^{3+} excited states.

Text:
The insets in Figures 3a,b show plots using Eq. 2 for the temperature dependence of emission intensity and the value $E = 1326 \pm 75$ cm^{-1} is obtained in Figure 3a, with a poorer fit to the scattered dataplot in Figure 3b.

Comment 4: The authors discussed the two states back energy transfer in the main text and supplementary information, but it is nice also to include the fitting graph to demonstrate that the equation is not well fitted.

Response: Thank you for the suggestion. We have included the graphs of the two state back energy transfer in the Supporting Information to demonstrate the poor fits of this equation with the inclusion of back energy transfer to the $^5D_1$ excited state.

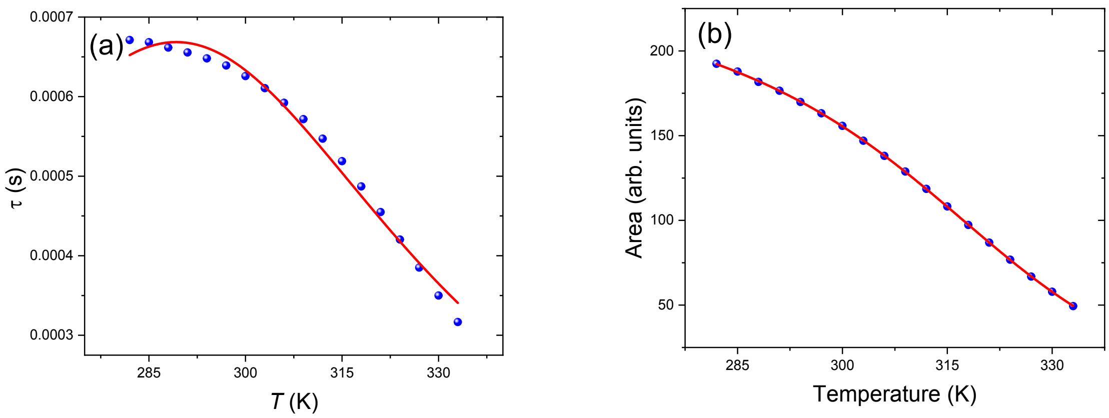

Figure S6. Fitting of **EuPhen(TTA)$_3$** lifetime and intensity data *vs* temperature by two barrier model.

The fit to (a) with 4 free parameters is:

$$\tau(T) = \frac{1}{(-4.2E3 \pm 1.3E4) + (7.4E6 \pm 2.0E6)\exp\left(-\frac{(1746 \pm 0)}{kT}\right) + (3.9E2 \pm 3.3E3)\exp\left(-\frac{(-499 \pm 1042)}{kT}\right)}$$

where $k = 0.6950536$ cm^{-1} K^{-1}, with $R_{adj}^2 = 0.98699$.

The fit to (b) with 4 free parameters is:

$$\frac{I(T)}{241 \pm 4} \approx \frac{1}{1 + (1703 \pm 210)\exp\left(-\frac{(1746 \pm 0)}{kT}\right) + (1.2E12 \pm 6.0E12)\exp\left(-\frac{(6178 \pm 1177)}{kT}\right)}$$

where $k = 0.6950536$ cm^{-1} K^{-1}. Although $R_{adj}^2 = 0.99997$ the parameter 1.2E12±6.0E12 is not meaningful.

Reviewer: 2

Recommendation: This paper is publishable subject to minor revisions noted. Further review is not needed.

Comments:
This manuscript entitled: "Thermally Activated Photophysical Processes of OrganoLanthanide Complexes in Solution" focuses on the mechanisms of thermal quenching properties for different Ln3+ ions of complex LnPhen(TTA)$_3$. They look deep into the energies from the back-transfer model for Ln = Sm, Nd and Yb cannot be linked to the ligand or charge transfer states. When Ln = Sm3+ exhibits quenching of intensity and lifetime with increasing temperature. On the other hand, Ln = Nd, Yb exhibit intensity quenching and lifetime lengthening with increasing temperature. This work is quite interesting, but it remains something that needs to be explained and discussed more clearly. We suggest that this paper be minor revised before publishing in The Journal of Physical Chemistry Letters.

Comment 1: In this article, the authors reported the thermally activated photoluminescence properties of organo-lanthanide complexes by using different lanthanide ions. However, the readers may lose focus because of the various effect of temperature analysis descriptions.

Response: Thank you for your suggestion. We have omitted the superfluous description of Sm^{3+} lifetime in the text in order make the manuscript more focused.

Comment 2: Energy level diagrams for the lanthanide ions have been marked in figure S1. However, some energy levels do not appear in this figure which may confuse the readers. Please mark all the energy levels clearly.

Response: Thank you for your suggestion. We have included a more detailed version of the energy level diagram of lanthanide 3+ systems in Figure S1.
